# Coordination of sustainable financing for evidence-based youth mental health treatments: protocol for development and evaluation of the fiscal mapping process

**DOI:** 10.1186/s43058-021-00234-6

**Published:** 2022-01-04

**Authors:** Alex R. Dopp, Marylou Gilbert, Jane Silovsky, Jeanne S. Ringel, Susan Schmidt, Beverly Funderburk, Ashley Jorgensen, Byron J. Powell, Douglas A. Luke, David Mandell, Daniel Edwards, Mellicent Blythe, Dana Hagele

**Affiliations:** 1 Department of Behavioral and Policy Sciences, RAND Corporation, 1776 Main Street, Santa Monica, CA 90401 USA; 2 Department of Pediatrics, Division of Developmental and Behavioral Pediatrics, University of Oklahoma Health Sciences Center, 940 NE 13th Street Suite 4900, Oklahoma City, OK 73104 USA; 3 Department of Economics, Sociology, and Statistics, RAND Corporation, 1776 Main Street, Santa Monica, CA 90401 USA; 4 Center for Mental Health Services Research, Brown School and School of Medicine, Washington University, Campus Box 1196, One Brookings Drive, St. Louis, MO 63130 USA; 5 Brown School, Washington University, Campus Box 1196, One Brookings Drive, St. Louis, MO 63130 USA; 6 Department of Psychiatry, University of Pennsylvania, 3535 Market Street, 3rd Fl., Philadelphia, PA 19104 USA; 7 Evidence-Based Associates, 1311 Delaware Ave, Suite 637, Washington, DC 20024 USA; 8 NC Child Treatment Program c/o Center for Child and Family Health, 1121 W, Chapel Hill St. Ste. 100, Durham, NC 27701 USA

**Keywords:** Youth mental health services, Evidence-based treatment, Financing strategies, Sustainment, Strategic planning, Tailored implementation strategies

## Abstract

**Background:**

Sustained delivery of evidence-based treatments (EBTs) is essential to addressing the public health and economic impacts of youth mental health problems, but is complicated by the limited and fragmented funding available to youth mental health service agencies (hereafter, “service agencies”). Strategic planning tools are needed that can guide these service agencies in their coordination of sustainable funding for EBTs. This protocol describes a mixed-methods research project designed to (1) develop and (2) evaluate our novel fiscal mapping process that guides strategic planning efforts to finance the sustainment of EBTs in youth mental health services.

**Method:**

Participants will be 48 expert stakeholder participants, including representatives from ten service agencies and their partners from funding agencies (various public and private sources) and intermediary organizations (which provide guidance and support on the delivery of specific EBTs). Aim 1 is to develop the fiscal mapping process: a multi-step, structured tool that guides service agencies in selecting the optimal combination of strategies for financing their EBT sustainment efforts. We will adapt the fiscal mapping process from an established intervention mapping process and will incorporate an existing compilation of 23 financing strategies. We will then engage participants in a modified Delphi exercise to achieve consensus on the fiscal mapping process steps and gather information that can inform the selection of strategies. Aim 2 is to evaluate preliminary impacts of the fiscal mapping process on service agencies’ EBT sustainment capacities (i.e., structures and processes that support sustainment) and outcomes (e.g., intentions to sustain). The ten agencies will pilot test the fiscal mapping process. We will evaluate how the fiscal mapping process impacts EBT sustainment capacities and outcomes using a comparative case study approach, incorporating data from focus groups and document review. After pilot testing, the stakeholder participants will conceptualize the process and outcomes of fiscal mapping in a participatory modeling exercise to help inform future use and evaluation of the tool.

**Discussion:**

This project will generate the fiscal mapping process, which will facilitate the coordination of an array of financing strategies to sustain EBTs in community youth mental health services. This tool will promote the sustainment of youth-focused EBTs.

**Supplementary Information:**

The online version contains supplementary material available at 10.1186/s43058-021-00234-6.

Contributions to the literature

- Approaches are needed that support financing the implementation and sustainment of evidence-based youth mental health treatments.
- This project will develop and evaluate the fiscal mapping process tool, which guides strategic planning to financially sustain evidence-based treatments in youth mental health service agencies.
- Results from the Delphi consensus process, comparative case studies, and participatory modeling will help refine the tool to better reflect agency and stakeholder needs, while preparing for future large-scale evaluation of the tool.
- Using the fiscal mapping process to support sustainable funding for youth evidence-based treatments may help achieve clinical, public health, and economic benefits.

## Background

One in 5 children [1] and 1 in 2 adolescents [2] experience a mental health problem annually, leading to considerable distress and impairment with an associated economic burden of $247 billion [3]. Research has identified evidence-based treatments (EBTs) that show clinical and cost-effectiveness for youth mental health outcomes [4–9] and can be economically feasible to implement [10–12], offering an important but underused way to improve quality of care. Addressing the societal impact of youth mental health problems requires that US mental health service systems offer EBTs widely and consistently [3, 13, 14]. Although many youths with mental health problems receive some treatment [15], service providers often offer treatments of limited or unknown effectiveness [16–19]—especially to youth from marginalized racial and ethnic groups [20–22].

One way to address this research-practice gap is improved implementation—the adoption and integration of EBTs in clinical service settings [23]. EBT implementation requires considerable financial resources, and limited and fragmented funding is one of the most-cited barriers to successful implementation processes and outcomes [24–28]. Ongoing investments also are needed to promote sustainment [29–31], defined as continued use of an EBT with ongoing program and population benefits [32, 33]. Without sustained use, the public health impact of EBT implementation is limited [34], yet many youth-focused EBTs are difficult to sustain [35–38]. In response, our team is developing and evaluating a strategic planning tool for the financial sustainment of EBTs in youth mental health services: the fiscal mapping process.

### Strategic planning to support EBT sustainment

The underlying premise of the fiscal mapping process is that the financial sustainment of EBT delivery requires youth mental health service agencies (hereafter, “service agencies”) to collaborate with their stakeholder partners [39–41] in order to navigate the complex, multi-level, and dynamic factors influencing sustainment [30, 38, 42, 43]. In the USA, service agencies include a variety of publicly and privately operated organizations (e.g., community mental health centers, hospitals, private organizations, children’s advocacy centers [44]). Service agencies’ stakeholder partners include (a) various third-party funding sources, including federal, state, and county agencies; public and commercial health insurance plans; and private foundations; and (b) intermediary organizations [45] that offer EBT implementation guidance and support to providers, for example, through expert training and supervision/consultation. It should be noted that third-party payors cover 87% of all US health care expenditures and, therefore, substantially influence service provider activities [46, 47].

Sustainable funding sources are not readily available for many activities that are essential to high-quality EBT delivery and commensurate reductions in youth mental health problems ﻿[43, 48–51]. Without funding, service agencies find it difficult to manage expenses for training and supervision/consultation, monitoring outcomes and EBT fidelity/adaptations, case management and care coordination, required resources and materials, and family- or group-based services [17–19, 24]. Support for direct service delivery traditionally comes from program budgets [38, 52, 53] and fee-for-service payments [54, 55], and these funds are often too limited to cover EBP delivery costs, let alone sustainment activities.

With the goal of promoting effective collaboration among US youth mental health service stakeholders, we grounded the fiscal mapping process in the Public Health Sustainability Framework [29, 56], which is comprised of eight core domains of sustainment capacities. The central domain in the framework is strategic planning (defined as processes that guide a program’s directions, goals, and strategies), which coordinates the other seven domains into an outcome-oriented plan. Although the fiscal mapping process focuses on the funding stability domain, our approach is informed by abundant evidence that funding stability relies on strategic planning capacities [29, 56–58]. Indeed, an agency may need to focus on building capacities in other domains—such as partnerships, political support, or communications—before funding stability is possible.

The importance of strategic planning to financial sustainment of EBTs is reinforced by theoretical work on the financing of public and non-profit private service organizations, showing that organizational success depends on the ability to identify and secure resources through diverse revenue streams (i.e., Resource Dependence Theory [25, 59, 60]) and that resource obtainment is influenced by relationships with the individuals and organizations that control those resources (i.e., Open Systems Theory [61, 62]). Numerous observational studies describe how service agencies often must engage in “creative financing” involving coordination of multiple funding sources to sustain EBTs [37, 48, 63, 64].

### Tailored selection of financing strategies as a solution

Strategic planning requires a sufficient understanding of the options available to achieve a goal or solve a problem. Implementation strategies are methods or techniques used to enhance implementation and/or sustainment [65]; various efforts are underway to compile and describe these strategies [66–68]. In one effort, a national group of implementation and financing experts identified and defined 23 financing strategies [69] that can support EBT implementation and/or sustainment in behavioral health systems. Example strategies included increased fee-for-service reimbursement, contracts for EBTs, and cost-sharing. This comprehensive compilation of financing strategies offers a foundation for the fiscal mapping process.

A catalog of EBT financing strategies is helpful, but insufficient, when selecting the optimal combinations of strategies necessary to sustain a particular EBT. Increasingly, implementation science emphasizes “tailored selection” [70–72] whereby various strategies are considered, then matched to the goals, needs, and constraints of a given implementation effort. Evidence to date suggests that tailored strategies promote implementation and health outcomes better than non-tailored strategies [70, 72]. Methods of tailoring implementation strategies are in their infancy, but implementation experts [71, 73] recently identified Intervention Mapping [74, 75] as showing promise for pragmatically selecting implementation strategies.

Intervention mapping is a well-specified, multi-step method for developing interventions, or implementation strategies [71, 73, 76], based on theory, research evidence, and stakeholder perspectives. The use of intervention mapping to tailor implementation strategies has led to successful EBT implementation in both uncontrolled and controlled studies [77–79]. We are adapting this process as a multi-step, structured tool that guides youth mental health service agencies in strategic planning efforts to finance EBT sustainment—the fiscal mapping process. Table 1 outlines the proposed steps of the fiscal mapping process as derived from intervention mapping. Briefly, these steps involve (1) identifying resources needed for EBT implementation, (2) specifying funding objectives linked to those needs, (3) matching financing strategies to the funding objectives, (4) selecting and using the best-fit combination of financing strategy options to meet all objectives, and (5) monitoring financial viability over time. The goal of the fiscal mapping process is to help service agencies select the optimal combination of strategies for financing their EBT sustainment efforts (within their existing constraints).

**Table 1:** Proposed steps of the fiscal mapping process, as adapted from intervention mapping

Step	Intervention mapping steps	Fiscal mapping process steps
1. Needs assessment	Identify determinants (barriers and facilitators) of implementation/sustainment	Identify resources needed for implementation/sustainment and determinants for those resources
2. Matrices	Specify program objectives and link those objectives to determinants	Specify funding objectives and link to determinants of needed resources for implementation/sustainment
3. Theory-based methods	Specify feasible, available implementation strategies matched to objectives	Specify feasible, available financing strategies matched to funding objectives
4. Intervention, adoption, and implementation^a^	Select strategies that best address the determinants	Enact financing strategies that best address resources needed for implementation/sustainment
5. Evaluation plan	Monitor and evaluate progress	Monitor and evaluate financial viability

^a^Intervention mapping has six steps [74, 75], but the “intervention program” and “adoption and implementation” steps are redundant and can be combined when the intervention is an implementation strategy [71, 73]

### Current project

This project will develop and evaluate the fiscal mapping process with key stakeholder input from youth mental health service agencies and their funding agency and EBT intermediary partners. The development process involves stages of feedback and revision aimed at gaining consensus on key fiscal mapping process steps. We will evaluate the preliminary impact of the fiscal mapping process through pilot-testing with ten youth mental health service agencies.

Participating agencies will pilot-test the fiscal mapping process with one of two widely disseminated EBTs for high-priority youth mental health problems: disruptive behavior problems and traumatic stress. Both of these clinical concerns have high prevalence rates (10–20%) [2] and result in severe personal, societal, and economic consequences well into adulthood if untreated [80–82]. The EBTs are parent-child interaction therapy (PCIT) [83] and trauma-focused cognitive-behavioral therapy (TF-CBT) [84]. PCIT is an EBT for youth ages 2–7 with disruptive behavior problems and their caregivers. It focuses on parent skill training in conjoint caregiver-child sessions, emphasizing positive interaction skills and effective discipline skills. TF-CBT is an EBT for youth ages 3–18 with traumatic stress symptoms. It is an exposure-based treatment that focuses on processing the traumatic experience and correcting problematic trauma-related beliefs, with sessions typically divided into youth, caregiver, and combined portions. There is extensive evidence for the clinical and cost-effectiveness of PCIT [85, 86] and TF-CBT [11, 87, 88]. By pilot-testing with two EBTs, we sought to promote the generalizability of the resulting fiscal mapping process.

This project is situated at the critical intersection of strategic planning for EBT sustainment, financing strategies, and youth mental health services. We will bring together knowledge from these three areas to develop and evaluate the fiscal mapping process.

## Method

We followed the Standards for Reporting Implementation Studies [89] (StaRI; see Additional file 1) for describing our project. All procedures were reviewed by the RAND Corporation Institutional Review Board and determined to not constitute human subjects research (Protocol #2020-N0607); nevertheless, we will follow all ethical principles for the protection of human research participants to minimize any risk of harm.

### Research design

**Fig. 1:**
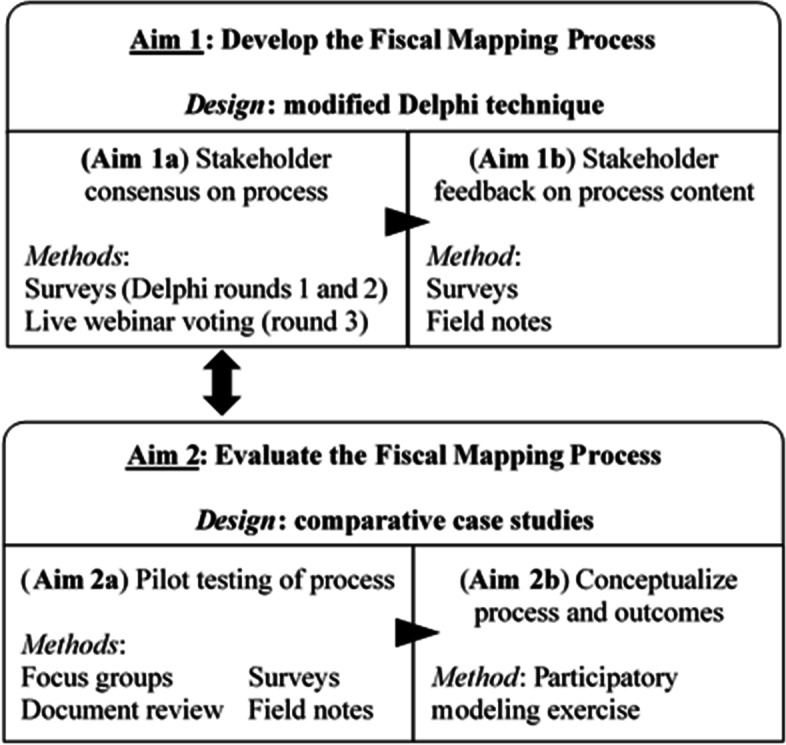
Overview of the research design for developing and evaluating the fiscal mapping process

Figure 1 summarizes our approach to developing (Aim 1) and evaluating (Aim 2) the fiscal mapping process. These aims have distinct designs, but will be completed concurrently over a 2-year period and inform each other throughout. Overall, we will use a mixed-methods [90] approach that examines the convergence between qualitative and quantitative data to provide an in-depth understanding of the fiscal mapping process.

Aim 1 is to develop the fiscal mapping process by adapting the intervention mapping process [74] and incorporating our compilation of financing strategies [69]. We will use a modified Delphi technique [91] to obtain formative stakeholder feedback. Delphi is a structured approach to group decision-making, and previous research has established its use for developing consensus about implementation strategies [66]. Sub-aims are to (1a) achieve consensus among our participants—through two web-based survey rounds followed by a round of live, virtual voting—on the key steps of the fiscal mapping process, while (1b) incorporating additional information into the financing strategy compilation to more fully inform strategy selection.

Aim 2 is to evaluate the preliminary impact of the fiscal mapping process. Our 2-year timeline is too short to observe sustainment trajectories, so we will instead focus on short-term factors related to EBT sustainment. Specifically, we will (2a) examine EBT sustainment capacities (e.g., for strategic planning) [29, 56] and outcomes (e.g., intentions to sustain) at the ten pilot-testing service agencies using a comparative case study approach [92, 93]. Each agency that pilot-tests the fiscal mapping process will be considered a case, and we will draw on multiple data sources (i.e., surveys, focus groups, document review, field notes) to compare and contrast experiences across agencies. Following pilot-testing, participants will contribute to a conceptual model of fiscal mapping’s process and outcomes through a participatory modeling exercise [94].

### Project timeline

The project began in February 2021, focusing first on recruitment and developing the initial fiscal mapping process prototype. When we completed this protocol in October 2021, we had finished recruitment and were conducting initial training with participating agencies; pilot-testing and data collection will take place over the subsequent 12 months. We will iteratively analyze data and incorporate it into the fiscal mapping process throughout pilot-testing, with the goal of finalizing the tool by the end of the project period (January 2023). This timeline coincides with the COVID-19 pandemic, but all project activities were planned to be conducted virtually which helped to minimize disruption.

### Participant and site recruitment

We have recruited 48 expert stakeholder participants, representing key roles in US youth mental health services, and we will engage them in all phases of the project. Stakeholder involvement is critical to producing research evidence relevant to those who deliver and fund EBTs [95–98]. Our recruitment plan is grounded in comparative case study methods [92, 93], using rigorous sampling to maximize the representativeness of small samples when random sampling is not feasible or effective [99, 100]. The cases are the ten service agencies, each represented by service agency representatives and their EBT intermediary and funding agency partners. Experts recommend recruiting approximately ten cases for subtle between-case comparisons [93], and representing multiple perspectives from each case [92]. For our sample, each agency will contribute up to 3 participants per stakeholder group. The resulting sample will allow us to use a variety of research methods, including—but also well beyond—case study methods.

#### EBT intermediary representatives

To begin recruitment, members of the research team nominated EBT intermediary organization representatives with expertise in the high-fidelity implementation and sustainment of PCIT or TF-CBT. We met our goal of recruiting 12 intermediary representatives; of the 12 enrolled intermediaries, five had expertise in PCIT, four in TF-CBT, and three in both models.

#### Youth mental health service agencies (cases)

Using snowball sampling [99, 100], intermediary representatives nominated service agencies with whom they worked to implement PCIT or TF-CBT in the past 5 years. We invited those agencies to apply to join the project and enclosed a detailed information guide about the project with each invitation. Our nomination and application process collected detailed quantitative and qualitative data about each agency from three stakeholder groups, which is ideal for rigorous case selection [100, 101] and will guide later comparative case study analyses. We received 45 service agency nominations and an additional six referrals from the nominated agencies, for a total of 51 nominees.

We found that youth mental health service agencies benefitted from technical support prior to their submitting an application. The principal investigator often met with agency representatives to engage them in the project and discuss key decisions, such as which service agency representatives should participate or which EBT would most benefit from the fiscal mapping process. We used purposive sampling [93, 99, 100] to prioritize cases for recruitment that provided a representative range of agencies, allowing for useful comparisons within and across our two EBT models of interest while providing adequate representation of service agency and funding agency participants. We recruited cases based on important characteristics of EBTs (e.g., use of PCIT vs. TF-CBT vs. both, use with racial/ethnic minority and low-income populations), agencies (e.g., type of agency, rural/urban service area, size), and funding contexts (e.g., state/region, service-funding agency partnerships). To ensure a clear focus on sustainment, agencies were required to have fully implemented the EBT of focus with at least one clinician.

In the application, agencies also contributed to our snowball sampling recruitment approach by nominating stakeholders involved in their EBT sustainment efforts to participate, including representatives from the service agency and from partner funding agencies. We then followed up with nominated individuals to verify their interest in participating (prior to finalizing an agency’s selection) and to gather demographic information.

Ultimately, 12 agencies submitted applications to join the project, of which ten 10 were selected to pilot-test the fiscal mapping process. Four of the participating agencies chose to focus on PCIT for the fiscal mapping the pilot test and the other six to focus on TF-CBT. The two agencies that applied but were not selected both had difficulty identifying service agency and/or funding agency representatives with the capacity to participate in the project (i.e., nominees from the application did not follow through with enrollment).

#### Youth mental health service agency representatives

Service agencies nominated personnel who had expertise and oversight regarding the financial aspects of EBT implementation and sustainment at the agency. We sought to recruit at least 18 service agency representatives; we found that nominated representatives were typically willing to participate once their service agency had committed and ultimately enrolled 24 service agency representatives. Most were in an agency leadership role (e.g., CEO, Chief Financial Officer, Vice President), a clinical administration role (e.g., clinical director, program supervisor), and/or a financial administration role (e.g., grants administration, development officer).

#### Funding agency representatives

Service agencies also nominated representatives from funding agencies that supported their EBT of focus in the past 5 years. Although we sought to recruit 18 funding agency representatives, service agencies reported it was challenging to identify funders who were willing to participate in this study. For example, some funding agencies had policies that precluded staff participation in research. Therefore, we concluded recruitment after enrolling 12 funding agency representatives, as this was equivalent to the number of intermediary participants and (given the higher-than-expected service agency representative enrollment) achieved the overall recruitment goal of 48 participants. The funding agency representatives came from a diverse range of organizations including state and tribal agencies, private foundations, and managed care.

### Pilot-testing activities

Pilot testing will provide service agency representatives with hands-on experience that can inform ongoing refinements of the fiscal mapping process. As a supplement to StaRI, here we follow the Template for Intervention Description and Replication [102] (TIDieR; see Additional file 2) when describing the fiscal mapping process and associated activities.

#### Fiscal mapping process tool

The research team created an initial prototype of the fiscal mapping process (version 1.0) for pilot-testing. The prototype format is an Excel workbook, and it is structured to clearly indicate what information should be entered to complete each step, but also flexible enough to accommodate agencies’ varied strategic planning goals and capture important contextual factors in each step. After specifying the focus of a given fiscal map (EBT, sites, etc.), the user completes the five fiscal mapping process steps: (1) resources needed, (2) funding objectives, (3) financing strategies, (4) fiscal map of EBT, and (5) monitoring plan (see Table 1). A resource tab accompanies each step with other materials useful for completing the step. For example, Step 1 resources include information about EBT time and cost models that help identify resource needs [103] and Step 3 resources summarize the aforementioned compilation of 23 financing strategies for behavioral health [69]. Each resource tab also includes a completed example of the associated step with a hypothetical service agency.

#### Initial training

We will provide a 3-h virtual training to the representatives from each pilot-testing service agency via Microsoft Teams. The agenda includes (a) introductions and project overview (30 min); (b) step-by-step instructions for using the fiscal mapping process, including ample hands-on discussion about completing the tool’s steps for the service agency (2 h); and (c) plans for coaching calls and data collection activities (30 min); regular breaks are included. We will promote engagement in the training through a practical, applied focus that allows agency representatives to leave training with an in-progress fiscal map and concrete next steps for using the tool. We will video-record each training session and give the service agency representatives access to the recording if desired. Two coaches (the principal investigator and project manager) will lead trainings for 5 agencies each; the other coach will attend to provide technical support and record detailed field notes. Both coaches have training in mental health service delivery (clinical psychologist and social worker, respectively) and EBT implementation.

#### Monthly coaching

To facilitate the use of the fiscal mapping process, each coach will provide monthly coaching sessions for 1 year with the service agencies for which they led training. Coaching sessions will be brief (~ 15 min per month) and focus on answering the service agency representatives’ practical questions about applying the fiscal mapping process. Prior to each coaching call, the coach will send a structured email inquiry asking representatives to specify (a) which fiscal mapping process steps they have worked on; (b) key areas they wish to prioritize for coaching, such as working toward completion of certain steps or deciding how to share conclusions with stakeholders; and (c) any desired modifications to the session format, like extending the session length or inviting stakeholders to join. The coach will also be available for as-needed consultation outside of the scheduled coaching calls; thus, rather than limiting coaching to 15 min, the use of this brief model provides a sustainable way to maintain monthly coach-agency contact for the duration of pilot-testing. Coaches will record field notes about the frequency, length, modality, and content of each coaching contact in a detailed logbook.

#### Plans to address adaptation and fidelity

Throughout the pilot-testing year, we will incorporate feedback from the 48 stakeholder participants into refinements of the fiscal mapping process. If there are major changes to the tool (Version 2.0, 3.0, etc.), then we will re-distribute it to participating agencies and provide additional guidance or training as needed. Thus, we will initially prioritize the adaptability of the fiscal mapping process while we incorporate stakeholder perspectives into the tool. Over time, we will develop a fidelity checklist of core fiscal mapping process steps that can be used by coaches as well as guide fidelity assessments for subsequent evaluations of the strategy.

### Data collection activities and measures

We will collect a mix of quantitative and qualitative data from the expert stakeholder participants for all project aims (see Fig. 1). Data will be collected using secure web-based programs: SelectSurvey for surveys and Microsoft Teams or Zoom.gov video-conference for the focus groups, webinar, and training/coaching activities. We will not collect personally identifiable information; participants will assign each participant a unique, anonymous identification number to identify their data. Table 2 provides a summary of each data collection activity, including the timeframe, measures used, participants involved, compensation amount, and relevant aims.

**Table 2:** Data collection activities for developing and evaluating the fiscal mapping process

Activity	Measures	Participants	Time	Compensation	Relevant aims^a^
Web survey #1 (months 1–3)			20–30 min	$30	
	Delphi Round 1	All, separately			Aim 1a
	Feedback on compilation of financing strategies^b^	All, separately			Aims 1b, 2a
	ICS, AFSS	Service agency reps			Aims 1b, 2a
Web survey #2 (months 6–9)		All, separately	20–30 min	$30	
	Delphi Round 2				Aim 1a
	Ratings of characteristics of financing strategies from compilation^b^				Aims 1b, 2a
Focus groups (months 4–5, months 10–11)			60 min	$50 per group	Aim 2a
	Semi-structured protocol	All three types together			
	PSAT, PRESS	All, separately			
	Intentions to sustain	Service agency reps			
	Documents for review	Service agency reps			
Webinar			Up to 120 min	$100	Aim 2b
	Delphi Round 3 (live voting)	All project participants			
	Participatory modeling exercise	together			
Field notes (training notes, coaching log, etc.)		Service agency reps	As relevant	n/a	Aims 1b, 2a

*Note*. ^a^See Fig. 1 for details of the project aims and how they relate to each other. ^b^Refers to the previously published compilation [69] incorporated into the fiscal mapping process. *AFSS* Agency Financial Status Scales [104], *ICS* Implementation Climate Scale [105], *PSAT* Program Sustainability Assessment Tool [56], *PRESS* Provider REport of Sustainment Scale [106]

#### Surveys

The modified Delphi [91] (Aim 1a) will begin with two rounds of feedback on the fiscal mapping process via online surveys administered 6 months apart. Each online survey will provide (a) a detailed description of each step of fiscal mapping; (b) a text box for comments, concerns, or proposed changes to each description; and (c) a text box to offer additional or alternative steps for the fiscal mapping process.

We will also incorporate feedback into the compilation of financing strategies [69] (Aim 1b) through two follow-up surveys (one in each of the first two Delphi rounds). Service agency representatives will provide additional information about their agencies in these follow-up surveys to provide context for the feedback. In the first survey, the expert participants will review the compilation and provide (a) quantitative ratings of each strategy’s relevance to youth mental health services, (b) qualitative feedback on each strategy, and (c) suggestions for additional financing strategies. Service agency representatives will provide ratings, using validated scales, of the agency’s implementation climate (Implementation Climate Scale [105]) and financial status for EBT implementation (Agency Financial Status Scales [104]). In the second survey, participants will provide ratings of each strategy’s availability in their funding environment, level of suitability for funding different implementation activities, feasibility, and effectiveness. Service agency representatives will also rate each strategy’s contribution to their funding for EBT sustainment (percentage of total funding over the last 3 years).

Each survey (Delphi + follow-up) is expected to take approximately 30 min. Participants will receive a $30 electronic gift card for each completed survey.

#### Focus groups

About 3 months after each survey, we will conduct a virtual focus group with each service agency. A given focus group will include one service agency’s representatives; the funding agency representative(s) nominated by the service agency; and an intermediary with expertise in the EBT of focus for pilot-testing (ideally, but not necessarily, the intermediary who nominated the agency). During the focus group, participants will discuss the service agency’s experience with pilot-testing the fiscal mapping process and how using the tool has impacted EBT sustainment capacities (from the Public Health Sustainability Framework [29]; especially financial stability and strategic planning) and outcomes. The groups will also discuss key characteristics of the EBT, agency, and funding context that influence the fiscal mapping process. The coach who does not conduct the agency’s coaching sessions will lead their focus group (to avoid demand effects). Focus groups will be supported by a research assistant who will take detailed notes, and will be audio-recorded for later analysis.

Each focus group is expected to take approximately 1 h, and participants will receive a $50 electronic gift card as compensation. Afterwards, participants will complete a brief web-based survey rating (a) the agency’s capacity for sustaining the chosen EBT using the Program Sustainability Assessment Tool, a measure of Public Health Sustainability Framework domains [56]; (b) extent of EBT sustainment using the three-item Provider REport of Sustainment Scale [106]; and for service agency representatives only (c) intentions to sustain the EBT over the next year. The focus group audio-recordings will be transcribed, with any identifying information removed, and destroyed once the analysis is complete.

#### Document review

To provide additional insights into the use of the fiscal mapping process, we will also collect and review relevant documents, such as agencies’ draft or final fiscal mapping process tools or information obtained from EBT intermediary and funding agency partners that informed completion of the tool. This method can provide useful insights into complex systems-level processes when interpreted alongside other qualitative and quantitative data [107]. We will identify relevant documents during the focus group discussions and coordinate with service agencies to support sharing as much as they are comfortable (establishing data use agreements and secure file transfers as needed).

#### Webinar: consensus voting and participatory modeling

At the end of pilot-testing, we will invite all 48 participants to participate in a 2-h webinar. Two data collection activities will be completed during the webinar: consensus voting for the final Delphi round (Aim 1a) and a participatory modeling exercise (Aim 2b). The two fiscal mapping process coaches will serve as facilitators.

The final Delphi round will be a live voting and consensus process. The facilitators will present each step of the process for voting, with associated comments and alternative specifications (if applicable). We will use the US Senate benchmark for a supermajority to end debate (≥ 60%) [108] for indicating consensus, as in a prior Delphi for implementation strategies [66]. We will attempt to identify consensus on a step using approval votes (i.e., for all acceptable options) before moving on to “run-off” voting, as this is the most efficient and “sincere” (i.e., strategy-proof) form of voting [109]. If consensus is not reached after runoff voting, the original description of the step will be retained. Throughout voting, participants can make comments in the chat or virtually “raise their hand” to make verbal comments for 1 min at a time. We will keep a record of the webinar polls used to count votes.

In the second portion of the webinar, participants will complete a participatory modeling exercise in which they conceptualize the process and outcomes of fiscal mapping. Participatory modeling is a technique from systems science that guides a group of stakeholders through the creation of a conceptual model of systems structures [94]. The facilitators will guide participants’ identification of actors, activities, outcomes, and contextual factors involved in each step of the process and solicit ideas for how to best evaluate changes in these factors. We will use the whiteboard function to illustrate the participants’ conceptual model in real-time as the discussion proceeds. To help make the discussion more engaging, we will solicit feedback through diverse channels including webinar polls, word clouds, chat box (including an anonymous option), and annotation on the whiteboard.

We will video-record the entire webinar to allow for a detailed record of the activities. The recording will be destroyed once the analysis is complete. We expect the entire webinar will take approximately 2 h, and attendees will each receive a $100 electronic gift card.

#### Field notes

As noted previously, coaches will log detailed field notes during training and coaching activities. In addition to being useful for the coaching process, these notes can be analyzed later for research purposes. The content of field notes will be most relevant for capturing service agency feedback on the fiscal mapping process (Aim 1b) and offering another source of insights into agencies’ experiences with the process and its outcomes (Aim 2a).

### Analysis plan

Our analytic approach is grounded in mixed methods, which is standard practice for implementation research [90]. Mixed methods involve combining quantitative data (Delphi votes, standardized scales) and qualitative data (e.g., focus group notes and transcripts, open-ended survey responses, document review, field notes) to gain higher-level insights that would not be possible through the use of either approach in isolation.

#### Initial data processing

We will calculate descriptive statistics for quantitative measures. For qualitative data, we will use rapid content analysis [110, 111] to distill major themes from a given data source. Rapid content analysis is ideal for synthesizing actionable conclusions from qualitative data to inform implementation activities, and it can be applied to a variety of written data sources (including documents and logs [107]). Qualitative themes will be critical for interpretation, given that our small sample precludes complex quantitative analyses. We will also calculate internal consistency reliability for each scale and compare quantitative and qualitative results as a validity check.

#### Aim 1: development

We will organize the quantitative and qualitative survey data (from Aims 1a and 1b) into response matrices, which will guide team discussions about how to incorporate stakeholder feedback into the fiscal mapping process. The matrices represent a mixed-methods convergence function [90], where cells will summarize the overlap between qualitative and quantitative feedback across different dimensions (e.g., EBT models, stakeholder types) to help identify key priorities. For example, we might make refinements to the prototype by adding, removing, or refining the steps; we might also incorporate additional resources, including summaries of survey ratings on the financing strategy compilation. Ultimately, we will produce a well-specified fiscal mapping process with consensus on the key steps involved [91, 108].

#### Aim 2: evaluation

Our evaluation will primarily rely on the comparative case study approach [92, 93], synthesizing all available quantitative and qualitative data for in-depth insights into each case (i.e., youth mental health service agency that pilot-tested the fiscal mapping process). This approach involves creating descriptive summaries of the role of the fiscal mapping process in EBT sustainment capacities and outcomes at each agency, clearly identifying the contributions of different qualitative and quantitative measures to the conclusions drawn. We will then compare and contrast the ten pilot-testing agencies based on the key characteristics in the sampling plan. At various points in the analysis, a given pair of agencies may be grouped together or contrasted, depending on the characteristic being considered. We will also consider differences in perspective among the three stakeholder participant groups (service agency, funding agency, intermediary). Statistical power is limited, but we will examine if quantitative data follow expected contrasts and patterns over time, such as more effective use of fiscal mapping at agencies with higher and/or increasing strategic planning capacities. We will heavily leverage qualitative data to ensure accurate interpretation and maximize depth of understanding.

To complement our comparative case studies, we will analyze the participatory modeling exercise results to create an overarching conceptual model of the fiscal mapping process that can guide future evaluation. Following the webinar, the project team will review the exercise results and create a system dynamics diagram [94] representing the conceptual model that the participants generated. The system dynamics diagram will specify actors, activities, outcomes, and contextual factors for each step of the fiscal mapping process, providing a visual representation of the complex interactions and feedback loops involved in EBT financing decisions. For specified outcomes of each step, we will also note key indicators for evaluating success. Finally, we will use the conceptual model to expand the Public Health Sustainability Framework [29]—which describes key capacity domains but is silent on how to evaluate their impact—so that the framework can guide prospective evaluations of the fiscal mapping process and other approaches targeting EBT sustainment.

## Discussion

This study will generate a novel fiscal mapping process, an innovative tool that will help service agencies identify and coordinate financing strategies for sustaining youth EBTs. Our research process and outputs will integrate existing knowledge from strategic planning for EBT sustainment, financing strategies, and youth mental health services in a stakeholder-friendly format. By rigorously developing and evaluating a strategic planning tool for EBT sustainment strategies [70–72], this project has great potential to improve sustainment outcomes. This is a complex undertaking, but our mixed-methods approach will integrate qualitative and quantitative data (i.e., surveys, focus groups, document review, field notes, Delphi method, and systems science) into an in-depth, comprehensive understanding of the fiscal mapping process.

This work is a unique effort to consider the important role of financing systems within efforts to support EBT implementation and sustainment. Consideration of financing systems introduces many challenges to the use of implementation strategies, including the need to coordinate strategic planning efforts among service delivery and financing agencies. Agencies will navigate how to maximize their prospects for EBT sustainment through a balance of (a) cultivating a diverse range of reliable funding sources while (b) keeping the entire process feasible to manage. These efforts will almost certainly involve additional sustainment capacities from the Public Health Sustainability Framework [29], such as agencies’ communications with stakeholders or partnerships in their communities. In fact, service agencies may need to advocate for funders to offer new financing strategies before they can realistically cover EBT sustainment costs. Service agencies may find it useful to present their fiscal map to stakeholders when communicating around gaps in current funding and priorities for future support.

We anticipate that various audiences will be interested in the broader implications of the knowledge we generate about financing strategies, and the methodological advances that we bring to this area of study. Potential audiences include state and federal behavioral health administrators, policymakers, youth mental health treatment organizations, and researchers in fields like implementation, health policy, and public finance. In addition to sharing new understanding, we also view dissemination efforts as an opportunity to collaboratively generate further knowledge with additional stakeholders. We are particularly interested in understanding when and how the fiscal mapping process should be introduced to youth mental health service agencies. For example, there may be advantages and disadvantages to introducing the tool earlier versus later in the EBT implementation process, or in having the tool introduced by EBT intermediaries (e.g., trainers), a neutral third party, or funding agencies. To date, implementation research has paid little attention to how implementation efforts should integrate strategies that target both practice-specific capacities (e.g., knowledge, skills) and capacities that support EBPs generally, such as the fiscal mapping process. We expect that conversations with stakeholders will be the ideal first step in exploring such decisions. Moreover, as we continue to develop and evaluate the fiscal mapping process, we will seek funding and opportunities to incorporate feedback from youth and family stakeholders as well; although they are not envisioned as users of the fiscal mapping process, we believe youth and families should have a voice in setting the broader strategic priorities that agencies pursue.

Beyond the implications for EBT financing, this project will provide an innovative advance in implementation research methods by expanding the Public Health Sustainability Framework [29] for use in the evaluation of EBT sustainment strategies (see Aim 2b). This expansion is an important step toward evaluating the effects of implementation strategies on long-term sustainment and health outcomes in future work. Few implementation research frameworks currently focus on sustainment [30, 112], and even fewer were designed for evaluation purposes [113, 114]. Even more broadly, our research on financing strategies may produce useful insights into other strategies operating in the outer setting [26, 48, 115], such as policies mandating EBPs [17]. Better understanding of implementation strategies that can support sustainment capacities and address systems-level issues (like financing) promises to improve the implementation, sustainment, and ultimate public health impact of youth mental health EBTs.

In keeping with best practices in policy dissemination [116, 117], we will maximize the impact of our dissemination efforts through strategies such as framing the presentation of results in ways that highlight their relevance to various stakeholder audiences, or sharing our results with intermediary organizations (e.g., mental health advocacy organizations, National Association of State Mental Health Program Directors) that have trusted relationships with administrators and policymakers. We also plan to make the fiscal mapping process tool available for public use, should our findings suggest that youth mental health service systems would benefit.

We recognize that most results derived from this pilot study will be preliminary and exploratory. This is especially so because our 2-year timeframe is not adequate for examining long-term effects on sustainment. If our evaluation outcomes are promising, we anticipate following up with a large-scale, randomized implementation-effectiveness trial [118] to rigorously test the impact of the fiscal mapping process—and its mechanisms—on EBT sustainment and fidelity outcomes, while monitoring clinical outcomes (i.e., mental health symptoms). It will also be important to test the generalizability of the fiscal mapping process with multiple EBTs and with agencies not involved in its development, as the results of this project will be limited to sustaining PCIT and TF-CBT in youth mental health services. We anticipate further testing with youth mental health EBTs would be the next step, but may need to expand the fiscal mapping process into other service sectors (e.g., schools, child welfare, primary care) and populations (e.g., parents, prevention with at-risk populations) to impact youth mental health at a population level. Of course, additional development and evaluation work will be necessary to confirm whether the fiscal mapping process is beneficial in different contexts.

## Conclusions

In sum, this project will develop the fiscal mapping process and evaluate its promise for promoting the financial sustainment of EBTs within youth mental health service agencies. The goal throughout will remain to help direct resources where they are most needed to support effective practices and promote health—particularly among our society’s most vulnerable and under-resourced communities.

## Supplementary Information

**Additional file 1**: StaRI checklist for Fiscal MappingR0.

**Additional file 2**: TIDieR checklist for Fiscal MappingR0. 

## Data Availability

Data sharing is not applicable to this article as no datasets were generated or analyzed during the current study. In the future, we will share quantitative data collected using standardized scales through the National Institute of Mental Health Data Archive (https://nda.nih.gov/), a collaborative informatics system created by the National Institutes of Health to provide a national resource to support and accelerate research in mental health. Future publications will also detail other materials that may be shared, such as the fiscal mapping process tool and supportive documentation, as deemed appropriate based on our findings.

## Abbreviations

EBT: Evidence-based treatment
PCIT: Parent-child interaction therapy
StaRI: Standards for Reporting Implementation Studies
TF-CBT: Trauma-focused cognitive-behavioral therapy
TIDieR: Template for Intervention Description and Replication
